# A Novel Allele in the Promoter of *Wx* Decreases Gene Expression and Confers Lower Apparent Amylose Contents in *Japonica* Rice (*Oryza sativa* L.)

**DOI:** 10.3390/plants13050745

**Published:** 2024-03-06

**Authors:** Weijie Tang, Haiyuan Chen, Suobing Zhang, Jun Tang, Jing Lin, Xianwen Fang, Gaoming Chen, Yunhui Zhang

**Affiliations:** 1 Provincial Key Laboratory of Agrobiology, Institute of Germplasm Resources and Biotechnology, Jiangsu Academy of Agricultural Sciences, Nanjing 210014, China; 2 Zhongshan Biological Breeding Laboratory, Nanjing 210014, China; 3 State Key Laboratory of Crop Genetics & Germplasm Enhancement and Utilization, Jiangsu Collaborative Innovation Centre for Modern Crop Production, Nanjing Agricultural University, Nanjing 210095, China; 4 Jiangsu Co-Innovation Center for Modern Production Technology of Grain Crops, Yangzhou University, Yangzhou 225009, China

**Keywords:** *Wx*, promoter, allele, AAC, *japonica* rice, nitrogen

## Abstract

*Wx* is the key gene that controls amylose content (AC), and various alleles have been found in rice populations. *Wx^b^* is the major allele in *japonica* and produces moderate AC (15~18%). It was recently found that editing the promoter of *Wx* could produce a series of alleles that have different Wx activities. Although some studies have edited the promoter, few studies have focused on the natural variations in *Wx*. Here, we used the Rice3K database to investigate variations in the *Wx* promoter and found that the allele Wx1764178 (A/G) has a higher LD (linkage disequilibrium) with the two key SNPs (1765751, T/G; 1768006, A/C), which could produce different *Wx* alleles and influence AC, as reported previously. Further study showed that the Wx1764178 allele (A/G) is functional and influences the expression of *Wx* positively. Editing the A allele using CRISPR‒Cas9 produced 36 and 3 bp deletions and caused a decrease in the expression of *Wx*. The apparent amylose content (AAC) in the edited lines was decreased by 7.09% and 11.50% compared with that of the wild type, which was the *japonica* variety Nipponbare with *Wx^b^* and the A allele at 1764178, while a complementary line with the G allele showed a lower AAC than the A allele with no effect on other agronomic traits. The AAC of the edited lines showed a higher increase than that of the wild type (Nipponbare, *Wx^b^*) in low-nitrogen conditions relative to high-nitrogen conditions. We also developed a dCAPS marker to identify the allele and found that the G allele has widely been used (82.95%) in *japonica*-bred varieties from Jiangsu Province, China. Overall, we found a functional allele (Wx1764178, A/G) in the *Wx* promoter that could affect AAC in *japonica* cultivars and be developed as markers for quality improvement in rice breeding programs.

## 1. Introduction

Rice quality is a complex characteristic [1]. Amylose content (AC) is considered to be the key component that controls the eating and cooking quality (ECQ) of rice [2], and the *Wx* gene has been identified as the most important factor controlling AC [3].

*Wx* was first identified over 30 years ago [4], and the gene encodes granule-bound starch synthase 1 (GBSS1), which plays a role in amylose synthesis [5]. After being phosphorylated on the surface of starch granules, GBSS1 regulates the synthesis of amylose as oligomers [6]. Protein targeting to starch 1 (PTST1) and GBSS1 interact to facilitate the latter’s localization on the surface of starch granules [7]. Correct GBSS1 localization necessitates the C-terminal carbohydrate-binding module (CBM) of PTST1, and the mutation of CBM can result in GBSS1 persistence in the plastid stroma [8]. Altogether, GBSS1 plays an important role in starch biosynthesis, especially in amylose synthesis.

With regard to the use of the *Wx* gene, researchers have mainly focused on the identification of alleles that could produce different levels of AC. Through an analysis of *Wx* alleles, it was found that *Wx^lv^* is the ancestral allele of the rice *Wx* gene and can produce an AC of about 27% [9]. In 1998, two Japanese studies found that a single base mutation could alter the posttranscriptional level of *Wx* and that *Wx^a^* and *Wx^b^* were separated by that single base [10,11]. The *Wx^b^* allele is mainly found in *japonica* rice [12], and it produces a moderate AC ranging from 15% to 18% [11], while the AC of *Wx^a^* varieties is about 26%. Later, the mutated allele *Wx-mp* was characterized as being able to produce a relatively lower AC (~10%) than that of *Wx^b^* in rice [13]. Recently, the *Wx^mw^* allele was identified and was found to produce a lower AC of 14% compared with that produced by *Wx^b^* but a higher one than that produced by *Wx-mp* [14]. In comparison with looking for natural or mutated alleles, gene editing is a superior method for fine-tuning Wx activity. A previous study demonstrated that editing the promoter can regulate the expression of *Wx*, resulting in continuous changes in the AC in transgenic lines [15,16].

Nitrogen plays a pivotal role in AC regulation in rice, and AC tends to increase under low-nitrogen conditions [17,18]. The glutamine synthetase (OsGS1;1) enzyme is crucial for nitrogen use efficiency (NUE), and the alternative splicing of *OsGS1;1* affects the AC in rice [19]. Previous studies have found that *Wx* is the key gene controlling AC [11,20,21]. Different haplotypes exhibit varying phenotypes under 0, 1, and 2 nitrogen treatments. Raising the *Wx* function and nitrogen fertilization highly decreased the quality of food. Moreover, the application of nitrogen fertilizer reduced the AC in the rice grain. Achieving an optimal level of nitrogenous fertilization while maintaining good rice quality poses future challenges [17]. Therefore, it is vital that researchers identify the possible genetic sites that could be used to balance NUE and good quality.

With developments in rice sequencing, more populations have been sequenced [22,23]. Researchers have also identified genes associated with various traits like heading date, grain length, and amylose synthesis using natural populations [24,25,26]. In terms of amylose synthesis, a *bHLH* transcription factor was discovered by analyzing the starch structure using 244 germplasm [26]. Furthermore, an elite haplotype of the *bHLH* transcription factor has been identified. The alternative allele A→G of *bHLH* was used to characterize this diagnostic SNP (single-nucleotide polymorphism), which may be utilized in focused breeding to produce high-amylose rice in the *indica* subspecies [26]. With the sequencing of Rice3K, which contains the various subpopulations published in [27], more variations (more than 32 million) [28] and more TIPs (transposable element insertion polymorphisms) were found [29,30]. Meanwhile, this database has also provided an extensive analysis of haplotypes [31,32]. Overall, the Rice3K database offers valuable information on rice variations, enabling the identification of superior haplotypes for breeding purposes. In terms of *Wx*, it remains unknown whether SNPs in the *Wx* promoter have any function in regulating *Wx* expression as well as in affecting its expression under different nitrogen conditions.

In this study, we focused on using the Rice3K database to investigate variations in the *Wx* promoter that regulate *Wx* expression and apparent amylose content (AAC). Using this database, we examined *Wx* haplotypes in *japonica* and discovered a functional allele in the *Wx* promoter. This Wx1764178 allele (A/G) influences *Wx* expression, and editing the A allele reduces both *Wx* expression and AAC. Complementary assays confirmed that the G allele produces lower AAC (about 15.3%) compared with the A allele. Additionally, we observed that the edited lines exhibited more pronounced responses than the wild type under low-nitrogen conditions. Variation analysis using a wild rice panel revealed that the G allele originated from the wild type subspecies [33]. Altogether, we identified a functional allele in the *Wx* promoter that regulated *Wx* expression and the AAC in *japonica*.

## 2. Results

### 2.1. Two Crucial SNPs of Wx Have Strong LD and Are Clearly Differentiated between Temperate and Tropical Japonica

To investigate the haplotypes of *Wx* in *japonica*, we analyzed the SNPs of *Wx* in the Rice3K database. The results revealed that there were 11 haplotypes based on nine SNPs of the *Wx* gene in *japonica* (Table 1). Among these SNPs, two SNPs (1765761, T/G; 1768006, A/C) were found to be predominant in a higher number of haplotypes. These two SNPs were identified as key SNPs influencing the AC in rice [9]. Furthermore, our findings indicated a strong linkage disequilibrium (LD) (*r*^2^ = 0.84) between the two SNPs, with the TA combination primarily occurring in temperate *japonica* and the GC combination in tropical *japonica*. Previous studies have demonstrated that the TA allele leads to a lower AC, ranging from 15% to 18% [9], suggesting that the more precise regulation of the AC in temperate *japonica* subpopulations may be influenced by other variations in promoters or genes.

### 2.2. Analysis of the Wx Promoter in Japonica

**Figure 1:**
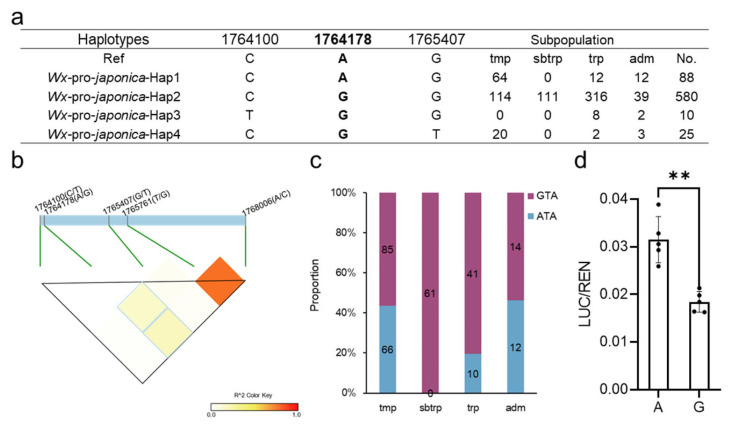
Analysis of the variations in the *Wx* promoter in *japonica*. (**a**) The haplotypes of the *Wx* promoter in *japonica* varieties. Ref: reference (Nipponbare); tmp: temperate; sbtrp: subtropical; trp: tropical; adm: admixed; No: number. (**b**) The LD among the three SNPs in the promoter and the two predominant SNPs in the gene. (**c**) The subpopulation distribution of the GTA and ATA (physical position: 1764178, 1765761, and 1768006). (**d**) Differences between the A and G allele (1764178) in the LUC assay. The data are presented as means ± SDs, n = 5. **: Student’s *t*-test, *p* < 0.05.

In an effort to explore the potential impact of promoter alleles on the expression of *Wx*, we conducted an analysis of the *Wx* promoter haplotypes of *japonica* using the Rice3K database. Our results revealed that four promoter haplotypes were present, with the majority of *japonica* (82.5%, 580/703) sharing the same promoter (Figure 1a, Table S1). Among these promoter haplotypes, Hap2 exhibited the highest proportion in subtropical *japonica*, while two unique haplotypes, Hap3 and 4, mostly emerged in tropical and temperate *japonica*, respectively. Although our findings suggested there are only three SNPs in the promoter of *japonica*, further exploration of the three SNPs was crucial for identifying the functional variations that fine-tune the expression of *Wx*.

### 2.3. The Wx1764178 Allele (A/G) in the Promoter Was a Functional Allele Affecting the Expression of Wx

To investigate whether the three SNPs in the promoter were functional, we calculated the LD between the promoter SNPs and the key SNPs (1765751, T/G and 1768006, A/C) in the gene. Encouragingly, the results indicated that the A/G allele (1764178) exhibited a higher LD with the two key SNPs in the *Wx* gene (Figure 1b), suggesting that the three SNPs (physical position as follows: 1764178, 1765716, and 1768006) co-segregated over a period. Furthermore, our investigation into the distribution of combinations in all *japonica* subpopulations revealed that the GTA and ATA showed nearly equal numbers in temperate and admixed *japonica*. In contrast, the GTA was the dominant combination in subtropical and tropical *japonica* (Figure 1c, Table S1). In a previous study, *Wx^b^* (TA) produced an AC ranging from 15% to 18% [9], and the Wx1764178 allele (A/G) could be involved in different haplotypes (ATA and GTA) based on the *Wx^b^* (TA) background. This result indicates that the GTA and ATA have different functions in *Wx* expression.

**Figure 2:**
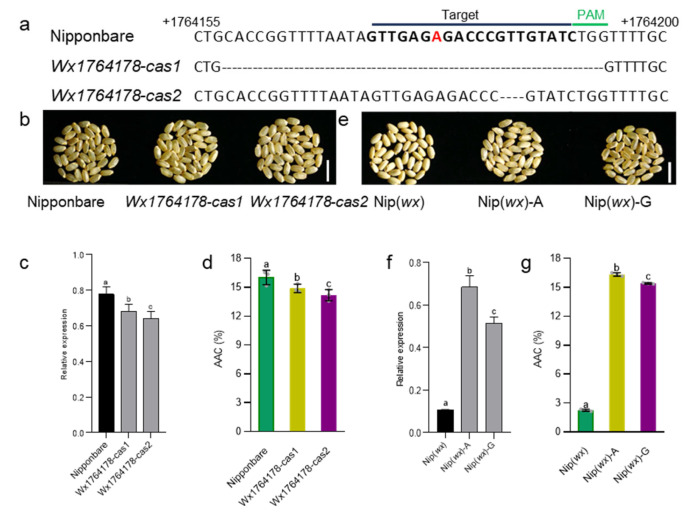
The transgenic validation of the Wx1764178 allele (A/G). (**a**) Targeted mutagenesis of Wx1764178 (A allele) with CRISPR-Cas9. The mutant alleles have 36 and 3 nucleotides deleted in *Wx1764178-cas1* and *cas2*. The red nucleotide represents the allele position 1764178. (**b**–**d**). The seed phenotype, *Wx* expression, and the AAC of the WT (Nipponbare) and the edited lines (*Wx1764178-cas1* and *cas2*); bar = 1 cm. (**e**–**g**) The seed phenotype, *Wx* expression, and the AAC of Nip(*wx*), as well as the complementary lines (Nip(*wx*)-A and Nip(*wx*)-G); bar = 1 cm. The data are presented as means ± SDs, n = 3. The significance of the differences was calculated using Duncan’s multiple range test. Different letters indicate significant differences, *p* < 0.05.

To determine whether the Wx1764178 allele (A/G) influenced the expression of *Wx*, we separately cloned the promoters of *Wx* with the A and G alleles. The results revealed that the A allele exhibited higher LUC activity compared with the G allele (Figure 1d), indicating that the Wx1764178 allele (A/G) is a functional allele that influences the expression of *Wx*. Subsequently, we employed CRISPR‒Cas9 to create promoter-edited lines. As a result, we obtained 16 transgenic lines, and 8 lines were edited in T0. In the T2 generation, we sequenced the target sequence of eight lines and found two homozygous mutations types that lacked the 36 bp and 3 bp (Figure 2a,b). The edited lines displayed a lower expression of *Wx* than that of the wild type as shown through qRT-PCR (Figure 2c), thereby leading to an AAC lower than that of Nipponbare, which was expected (Figure 2d). Additionally, complementary lines were constructed in Nip(*wx*), which was a variety with the null allele of *Wx*. As a result, the expression of the complementary lines being restored to the level of the wild type Nipponbare indicated that there was a copy in the complementary lines (Figure 2c,f). The results of the homozygous T2 lines also indicated that the expression of the A allele was higher than that of the G allele (Figure 2e,f), which was consistent with the results of the LUC assay. Furthermore, both lines with the A and G alleles exhibited a higher AAC than that of Nip(*wx*), with the line containing the A allele demonstrating the highest AAC (Figure 2g). These observations suggest that the Wx1764178 allele (A/G) is a functional allele that influences the expression of *Wx* and that the G allele could produce a lower AAC (by about 15.3%) than that of the A allele.

### 2.4. Analyzing the Effect of the Wx Expression Level on Agronomic Traits

**Figure 3:**
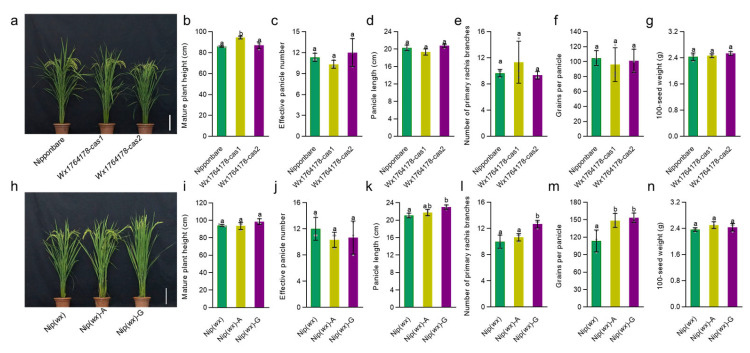
Analysis of the agronomic traits in the edited lines and complementary lines. (**a**) The phenotypes of the Nipponbare and edited lines, bar = 20 cm. (**b**–**g**) Comparison of the plant heights, effective panicle numbers, panicle length, number of primary rachis branches, grains per panicle, and 100-seed weight in the Nipponbare and edited lines (*Wx1764178-cas1* and *cas2*). (**h**) The phenotype of the Nip(*wx*) and complementary lines (Nip(*wx*)-A and Nip(*wx*)-G); bar = 20 cm. (**i**–**n**) Comparison of the plant heights, effective panicle numbers, panicle length, number of primary rachis branches, grains per panicle, and 100-seed weight in Nip(*wx*), as well as the complementary lines (Nip(*wx*)-A and Nip(*wx*)-G). The data are presented as means ± SDs, n = 3. The significance of the difference in the agronomic traits was calculated using Duncan’s multiple range test. Different letters indicate significant differences, *p* < 0.05.

Following this, we assessed whether any of the transgenic lines exhibited changes in agronomic traits such as plant height, effective panicle numbers, panicle length, number of primary rachis branches, grains per panicle, and 100-seed weight. While most traits showed no difference in the transgenic lines compared with the wild type (Nipponbare and Nip(*wx*)) (Figure 3a–g), the plant height of *Wx1764178-cas1* in the edited lines was higher than that of the wild type (Figure 3b). In the complementary lines, the agronomic traits of plant height, effective panicle number, and 100-seed weight showed no significant differences compared with Nip(*wx*) (Figure 3h–j,n), while the panicle length, number of primary rachis branches, and grains per panicle increased in the complementary line (Nip(*wx*)-G) (Figure 3k–m). These results indicated that the edited and complementary lines could produce a different level of AAC due to the A/G allele (1764178) and that this had no influence on other agronomic traits except for the plant height in the edited lines and grains per panicle in the complementary lines.

### 2.5. The Edited Lines Could Respond to Different Concentrations of Nitrogen

**Figure 4:**
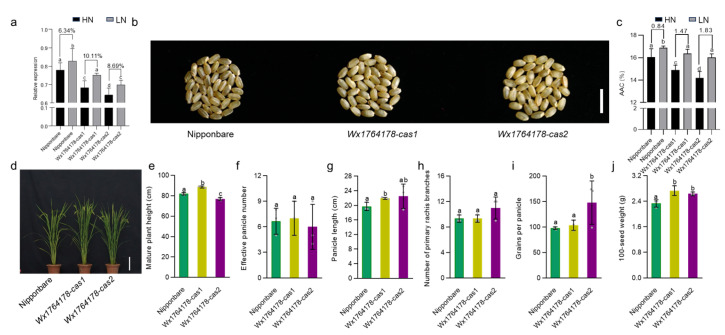
Analysis of the traits of the Nipponbare and edited lines under the low-nitrogen condition. (**a**) The *Wx* expression of the Nipponbare and edited lines (*Wx1764178-cas1* and *cas2*) under high- and low-nitrogen conditions. (**b**) The seed phenotype of the Nipponbare and edited lines (*Wx1764178-cas1* and *cas2*) under the low-nitrogen condition, bar = 1 cm. (**c**) The AAC of the Nipponbare and edited lines (*Wx1764178-cas1* and *cas2*) under high- and low-nitrogen conditions. (**d**) The phenotype of the wild type and edited lines under the low-nitrogen condition; bar = 20 cm. (**e**–**j**) Comparison of the plant height, effective panicle number, panicle length, number of primary rachis branches, grains per panicle, and 100-seed weight in the Nipponbare and edited lines (*Wx1764178-cas1* and *cas2*) under the low-nitrogen condition. HN: high-nitrogen condition; LN: low-nitrogen condition. The data are presented as means ± SDs, n = 3. The significance of the differences in *Wx* expression, AAC, and agronomic traits were calculated using Duncan’s multiple range test. Different letters indicate a significant difference, *p* < 0.05.

It should be noted that AAC could be altered due to the concentration of nitrogen during planting [34]. In order to investigate whether the edited lines had a different response to nitrogen, we subjected the edited lines to field conditions with high and low levels of nitrogen. The expression of *Wx* was found to decrease in the edited lines under both low- and high-nitrogen treatment compared with that of Nipponbare (Figure 4a). Low nitrogen increased *Wx* expression both in the Nipponbare and the edited lines. However, the extent of the increase was higher in the edited lines (Figure 4a). In order to investigate the influence of the nitrogen concentration on AAC, we detected the AAC in the different nitrogen conditions. Under the low-nitrogen condition, the change in the AAC in the edited lines increased by 1.47% and 1.83% in *Wx1764178-cas1* and *cas2* compared with the high-nitrogen condition, while the change was 0.84% in the wild type (Figure 4b,c). The assessment of the agronomic traits under low-nitrogen conditions revealed that the effective panicle numbers and the number of primary rachis branches and grains per panicle showed no significant differences between the wild type and edited lines (Figure 4d,f,h). The plant height, panicle length, and 100-seed weight were higher in *Wx1764178-cas1* (Figure 4e,g,j), while the grains per panicle and the 100-seed weight increased in *Wx1764178-cas2* (Figure 4i,j). These findings suggested that the edited lines exhibited a stronger response of *Wx* expression and AAC under the low-nitrogen treatment.

### 2.6. The Origin and Breeding Use of the Allele

**Figure 5:**
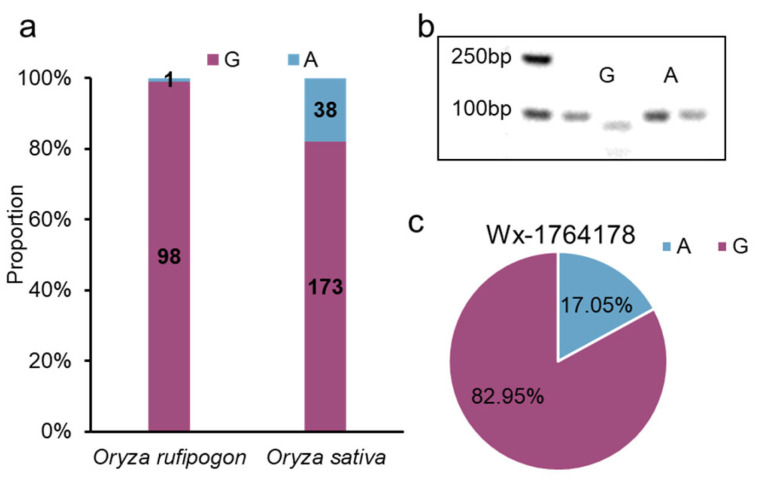
The origin and breeding use of Wx1764178 allele. (**a**) The distribution of Wx1764178 allele (A/G) in *Oryza rufipogon* and *Oryza sativa*. (**b**) The result of dCAPS marker of the Wx1764178 allele (A/G). (**c**) The distribution of Wx1764178 allele (A/G) in *japonica*-bred varieties from Jiangsu Province, China.

To gain insight into the origin of the alleles, particularly the G allele, we examined the variation in a wild rice panel and found that the G allele had a frequency of 98/99 in *Oryza rufipogon*, which was higher than that in *Oryza sativa* (Figure 5a). This indicated that this allele of the promoter originated from the wild species. Furthermore, we developed derived cleaved amplified polymorphic sequence (dCAPS) markers capable of detecting the allele (Figure 5b). Using this marker, we detected the allele in 88 *japonica*-bred varieties from Jiangsu Province, China, and found that the G allele had a higher frequency than that of the A allele (G allele: 73; A allele: 15) (Figure 5c, Table S2). In summary, this allele originated from wild rice, with the G allele being capable of decreasing *Wx* expression; it has been widely used in *japonica*-bred varieties from Jiangsu Province, China.

## 3. Discussion

### 3.1. Longer Sequence in the Promoter Should Be Studied Using a More Appropriate Approach

In a previous study, researchers used gene editing technology to produce novel alleles of *Wx*, with the promoter length being only −2310 from the ATG [16] in the *Wx^b^* background and only −2192 from the ATG in the *Wx^a^* background [15]. In our study, we identified a functional Wx1764178 allele (A/G) in the −2843 relative position from ATG, indicating the potential for discovering more SNPs and other polymorphic sites in the promoter beyond 2 K, and we investigated their function. This could aid in finding more novel alleles. On the other hand, we created edited lines that lacked either the 3 bp or 36 bp, and we could not prove whether or not the change in the expression of the edited lines was due to the deletion of neighboring nucleotides. In future studies, a more appropriate experimental approach like that of base editing would be better suited for proving the function of the Wx1764178 allele (A/G) [35].

### 3.2. Upstream Regulated Genes Could Be Investigated for Network Construction

In past studies, many alleles, including *wx*, *Wx^mp^*, *Wx^op^*, *Wx^mw^*, *Wx^a^*, *Wx*(*hp*), *Wx^in^*, *Wx^b^*, *Wx^lv^*, and *Wx^lv-w^*, were identified [9,10,11,13,36]. These natural alleles, based on variations in the gene, could produce a level of AAC ranging from ~2% to ~27%. Additionally, a previous researcher created more alleles in the *Wx* gene using gene editing technology [37]. In addition to the exploration of alleles in the gene, other alleles have been created in the promoter [15,16]. The regulation of *Wx* by transcription factors such as OsbZIP58 [38] and OsSMF1 [39] was also evaluated. In our study, we used the Rice3K database, which contains many variations in *Wx*, to identify novel alleles. The Wx1764178 allele (A/G) that we identified will increase our understanding of *Wx* alleles, especially the alleles in the promoter. However, we only focused on the validation of the allele, and its possible mechanism was not studied. Nevertheless, we found that the Wx1764178 allele (A/G) was located in a GAGA motif, which could be bound by GAGA-motif binding factors (GAFs). In rice, the *OsGBPs* family gene could recognize the GAGA motif, and *OsGBP1* and *OsGBP3* have higher expression in the panicle and affect the grain length [40]. The deletion in our edited lines may affect the recognition or binding by upstream transcription factors to decrease the expression of *Wx*. In future studies, the regulatory network could be investigated based on Wx1764178 and more genes may be found, which could be helpful for improving rice quality.

### 3.3. The Direct Connection between Nitrogen and the Allele Should Be Further Studied

Nitrogen is a factor affecting rice quality. It can increase yield, but it also decreases the ECQ of rice [18]. With the increase through nitrogen fertilizers, the AC of rice decreased [17]. Thus, we still know less about the relationship between Wx of the key controlling AC gene and nitrogen. In recent studies, the transcription factor *Nhd1* was shown to influence NUE [41], and the expression of *Nhd1* was shown to have a positive relationship with the ECQ of rice [42]. *Nhd1* can affect NUE and AC and provide a relation between nitrogen and *Wx* through transcriptional regulation. In our study, we used the edited lines of *Wx* and proved that it can respond more under different nitrogen conditions compared with Nipponbare. However, we only observed the phenotype of the edited lines and did not investigate the mechanism underlying the direct connection between nitrogen and the allele. The possible transcriptional regulation of this effect should be investigated in future studies.

### 3.4. The Allele Could Be a Target for Improving Rice Quality under Different Nitrogen Conditions

In this study, we planted the edited lines in different fields with different nitrogen conditions. The AAC alteration in the edited lines changed more than in the wild type, and the results showed that the edited lines responded more than the wild type. This allele could provide a potential candidate site for quality breeding under different nitrogen conditions in rice. Furthermore, these polymorphisms could provide a target position for gene editing. *Wx^b^* has been observed to produce a relatively lower AC (15~18%), but variations in the promoter could combine with variations in the gene (*Wx^b^*). Recently, researchers used high-efficiency multiplex promoter targeting (HMP) to generate novel alleles in the cis-regulatory regions of key genes and achieved a series of germplasms with quantitative variations in the heading date of rice [43]. In future studies, we could combine natural variations in populations with editing sites to create more efficient alleles for rice breeding.

## 4. Materials and Methods

### 4.1. Data Download and Haplotype Analysis

The SNPs of *japonica* (855 *japonica* varieties including 288 temperate, 112 subtropical, 372 tropical, and 83 japx *japonica*) were downloaded from the Rice3K database (snp-seek.irri.org, accessed on 18 April 2020) [27]. The reference is Os-Nipponbare-Reference-IRGSP-1.0. The SNPs in the gene and promoter (~3K from ATG) of *Wx* were analyzed. We downloaded 695 markers in total. We deleted the markers with no polymorphisms and for which polymorphic sites were heterozygous. The markers with missing positions >20 were also deleted from the analysis. Overall, we obtained 12 SNPs for further study. The variety information of Rice3K, including the subpopulation and country, was obtained from the website (snp-seek.irri.org, accessed on 18 April 2020). The allele of *Oryza rufipogon* was downloaded from the website (http://server.ncgr.ac.cn/RiceHap3/, accessed on 21 May 2022). The reference is IRGSP version 4.0, and the allele position is 1763179 on chromosome 6, while it is 1764178 on chromosome 6 in Os-Nipponbare-Reference-IRGSP-1.0. The haplotype and LD were analyzed using the software TASSEL 5 with default parameters [44].

### 4.2. Luc Assay

The promoter (~3 K sequence from the ATG, including the A/G (1764178) allele) was amplified. The fragments were cloned into the pGreen II 0800-*LUC* (P35Smini::LUC) vector (HindIII and EcoRI). *N. benthamiana* leaves were agro-infiltrated with *A. tumefaciens* EHA105 strains carrying the various combinations of the DNA construct (*Wx*-1764178-A:luc, *Wx*-1764178-G:luc). Leaves of *N. benthamiana* were harvested after 48 h of infiltration, and their luciferase activity was assayed. The *N. benthamiana* leaves were kept in the dark for 5 min after adding 1 mM luciferin to quench the fluorescence. Quantitative LUC activity was determined by a Microplate Luminometer (Promega, Madison, WI, USA). The primers are listed in Table S3.

### 4.3. Editing and Complementary Lines Development

The target of the edited lines was designed using the website cbi.hzau.edu.cn/cgi-bin/CRISPR (accessed on 20 October 2021). The T0 and T2 lines were sequenced using Sanger sequencing. The two homozygous lines were selected for expression detection and trait collection. *Wx1764178-cas1* and *cas2* lacked the 36 and 3 bp, respectively.

The complementary lines were constructed using different promoters (~3 K sequence including the A (1764178) allele or G (1764178) allele) and the *Wx^b^* gene, which was cloned from the Nipponbare variety. Both promoters were cloned into the pCUbi1390 vector *(HindIII* and BamHI). Then, the fragment of the *Wx^b^* gene was cloned into the pCUbi1390 vector (with the *Wx* promoters of different alleles) for construction of the complementary lines (Nip(*wx*)-A, Nip(*wx*)-G). The constructed vectors were transformed into Nip(*wx*), which has *wx* in the Nipponbare background [9]. The AAC in Nip(*wx*) was about 2%. The homozygous T2 lines were used for expression detection and trait collection. The copy number of the transgene in the transgenic rice plants was determined using real-time PCR according to Zhang et al. [17]. The primers are listed in Table S3.

### 4.4. Expression Profiling

To investigate the expression of the *Wx* gene in the wild type (Nipponbare and Nip(*wx*)), the edited lines, and the complementary lines, with the total RNA being isolated using an RNA prep Pure Plant Kit (Tiangen Biotech, Beijing, China) using the mature seeds. Then, ~1 μg of total RNA was reverse-transcribed into cDNA using a PrimeScipt^TM^ Reverse Transcriptase kit (Takara, Shiga, Japan, www.takarabio.com, accessed on 9 October 2023). Real-time PCR was performed in a real-time PCR machine (I-Cycle, Bio-Rad, Hercules, CA, USA), with each reaction containing 5 μL of first-strand cDNA, 5 μL 2 mmol L^−1^ gene-specific primers, and 10 μL SYBR Premix Ex Taq™ (Takara, Shiga, Japan, www.takarabio.com, accessed on 9 October 2023). The amplification conditions were 95 °C for 30 s followed by 40 cycles of 95 °C for 5 s and 60 °C for 34 s. The primers are listed in Table S3. The rice *UBQ* (*Os03g0234350*) gene was used as an internal control. Three biological replications were conducted.

### 4.5. Measurement of Amylose Content

AAC was measured following the method of Tan et al. (1999) [45]. Briefly, the rice grains were de-hulled and milled in duplicate using a miller based on the National Standards NY 147–88 (NY stands for the abbreviation of “Agricultural” in Chinese spelling). In a water bath set at 50 °C, precisely 25 mg of rice flour was gelatinized overnight in 2 mL of 1.0 N NaOH. After 10 min of boiling in the water bath, the solution was allowed to cool to room temperature. After removing the lipid from the cooled solution three times using 5 mL of butanol/petroleum ether (1:3), 1.5 mL of 0.4 N KI was added, and the mixture was stirred. To prepare the standard amylose solutions (5%, 10%, 15%, 20%, 25%, and 30%), pure amylose was dissolved in distilled water.

### 4.6. Phenotypic Characterization

All materials were planted at the Jiangsu Academy of Agricultural Sciences. The transgenic lines were treated under high- and low-nitrogen conditions (+N with 300 kg/ha N as well as 0 N with 0 kg/ha N). All materials under the high- and low-nitrogen treatments were sown on May 9th and transplanted on June 9th at the same time. The accessions were cultivated in a completely randomized block pattern with two replicates for the field experiments. There was a gap of 20 and 17 cm between rows and individuals, respectively. Three independent lines were chosen for agronomic trait (plant height, effective panicle numbers, panicle length, number of primary rachis branches, grains per panicle, and 100-seed weight) collection. The agronomic traits were collected in the mature stage.

### 4.7. Marker Design and Detection

The sequence was downloaded from Gramene (https://ensembl.gramene.org/Oryza_sativa/Info/Index, accessed on 14 July 2023). The dCAPs primers were designed using the website dCAPS Finder 2.0 (http://helix.wustl.edu/dcaps/, accessed on 14 July 2023). All DNA samples were extracted using the DNAsecure plant kit (Qiagen (Hilden, Germany), Cat. No. DP320, accessed on 13 October 2023). Amplification conditions were 95 °C for 5 min followed by 36 cycles of 94 °C for 30 s, 58 °C for 30 s, 72 °C for 30 s, and 72 °C for 5 min. The restriction enzyme *BamHI* was used for detecting different alleles of *Wx*-1764178. The primers are listed in Table S3.

## 5. Conclusions

Novel allele identification in *Wx* could provide more tools for rice breeders. Here, we identified a functional Wx1764178 allele (A/G) of *Wx* from an analysis of haplotypes using the Rice3K database. A LUC assay showed that the allele could affect the expression of Wx, and transgenic lines proved the change in expression and that it had an effect on AAC. Under different nitrogen conditions, the AAC of the edited lines changed more than that of the wild type. Finally, we found that this allele has been widely used in *japonica* breeding. Overall, our study increased the knowledge of variations in *Wx,* and this allele could be useful for *japonica* rice breeding.

## Figures and Tables

**Table 1:** The haplotypes of the *Wx* gene in *japonica* rice. Ref: reference (Nipponbare); tmp: temperate; sbtrp: subtropical; trp: tropical; adm: admixed; No: number.

Haplotypes	1765 637	1765 761	1765 979	1766 437	1766 522	1767 052	1768 006	1769 643	1770 223	Subpopulation				
Ref	C	T	G	C	G	C	A	C	A	tmp	sbtrp	trp	adm	No.
*Wx*-gene-*japonica*-Hap1	C	T	G	C	G	C	A	C	A	167	38	50	35	290
*Wx*-gene-*japonica*-Hap2	C	T	A	C	G	C	A	C	A	11	0	0	0	11
*Wx*-gene-*japonica*-Hap3	C	T	G	C	G	T	A	C	A	0	7	0	0	7
*Wx*-gene-*japonica*-Hap4	C	T	G	C	G	C	A	T	A	0	0	5	0	5
*Wx*-gene-*japonica*-Hap5	C	T	G	C	G	C	C	C	T	0	5	0	0	5
*Wx*-gene-*japonica*-Hap6	C	T	G	C	G	C	C	C	A	6	4	8	0	18
*Wx*-gene-*japonica*-Hap7	C	G	G	C	G	C	A	C	A	0	0	3	0	3
*Wx*-gene-*japonica*-Hap8	C	G	G	C	G	C	C	C	A	3	14	178	17	212
*Wx*-gene-*japonica*-Hap9	C	G	G	C	T	C	C	C	A	24	0	0	3	27
*Wx*-gene-*japonica*-Hap10	C	G	G	T	G	C	C	C	A	0	9	0	0	9
*Wx*-gene-*japonica*-Hap11	A	G	G	C	G	C	C	C	A	0	0	3	0	3

The green color represented two key SNPs in *Wx* gene.

## Data Availability

The datasets supporting the conclusions of this article are included within the article and Supplementary Materials.

## Supplementary Materials

The following supporting information can be downloaded at: https://www.mdpi.com/article/10.3390/plants13050745/s1.
